# Do Pollinators Contribute to Nutritional Health?

**DOI:** 10.1371/journal.pone.0114805

**Published:** 2015-01-09

**Authors:** Alicia M. Ellis, Samuel S. Myers, Taylor H. Ricketts

**Affiliations:** 1 Gund Institute for Ecological Economics, University of Vermont, 617 Main St., Burlington, VT 05405, United States of America; 2 Harvard School of Public Health, Department of Environmental Health, 401 Park Drive, Room 404-M, Boston, MA 02215, United States of America; 3 Harvard University Center for the Environment, 24 Oxford St, Room 307, Cambridge, MA 02138, United States of America; Central China Normal University, China

## Abstract

Despite suggestions that animal pollinators are crucial for human nutritional health, no studies have actually tested this claim. Here, we combined data on crop pollination requirements, food nutrient densities, and actual human diets to predict the effects of pollinator losses on the risk of nutrient deficiency. In four developing countries and across five nutrients, we found that 0 to 56% of populations would become newly at risk if pollinators were removed. Increases in risk were most pronounced for vitamin A in populations with moderate levels of total nutrient intake. Overall, the effects of pollinator decline varied widely among populations and nutrients. We conclude that the importance of pollinators to human nutrition depends critically on the composition of local diets, and cannot be reliably predicted from global commodity analyses. We identify conditions under which severe health effects of pollinator loss are most likely to occur.

## Introduction

Growing evidence indicates that human-induced changes to the environment may have widespread consequences for human health [1]–[4]. The decline of animal pollinators is one such change. Populations of both managed and wild pollinators are declining around the globe [5]–[7], and the importance of pollinators to farm productivity and economics is increasingly clear [8], [9]. Moreover, recent studies have estimated that pollinators are responsible for up to 40 percent of the world's supply of nutrients [10], and have shown that areas of pollinator importance can occur within countries of high micronutrient deficiency [11]. A common conclusion is that a decline in pollinator populations could have a “potentially drastic effect on human nutrition” [10].

**Figure 1:**
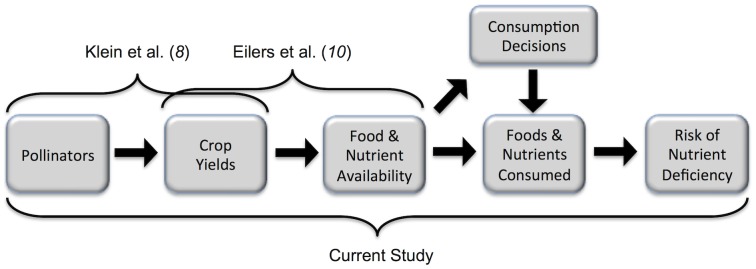
Conceptual framework of the influence of pollinators on risk of nutrient deficiency. Klein et al. [8] studied the first arrow and Eilers et al. [10] studied the second. Our study builds on these to examine the complete causal pathway.

Despite these estimates and the extensive media coverage of the issue (e.g, [12]), no studies to date have tested this nutrition claim empirically. While pollinators improve yields for crops that contribute nutrients to the food supply, the role that pollinated crops actually play in the nutritional health of individuals and populations remains unclear. Understanding this role requires additional information on actual diets, nutrient consumption, and baseline levels of nutrition (Fig. 1).

Micronutrient deficiencies are estimated to affect more than 1 in 4 people around the globe [13]. The “hidden hunger” associated with vitamin and mineral deficiencies affects individuals of every age and gender and can cause increased risk of maternal mortality, increased incidence of a variety of chronic and infectious diseases, reduced IQ, decreased work productivity, and increases in nutrient-specific diseases like goiter, night-blindness, and iron-deficiency anemia. Collectively, they are responsible for a large, global burden of disease from increased morbidity and mortality. Thus, if pollinators do, in fact, contribute to nutritional health, continued declines of pollinator populations could have drastic consequences for global public health.

Here, we present results of the first empirical test of how pollinators influence nutrient intake and risk of nutrient deficiency. We focused our analyses on children and women in developing countries, where high rates of malnutrition and limited access to nutrient supplements may make individuals more susceptible to the effects of pollinator declines, and analyzed five of the most important nutrients to global nutrition: vitamin A, zinc, iron, folate, and calcium [14], [15] (data on iodine, another globally important nutrient, were not available in the datasets we analyzed). Because deficiencies in young children may be most important in determining long-term health, cognitive abilities, and survival [16] and because results were similar for women and children, we present mainly results for children aged one to three years old. Results for all age categories can be found in the supplemental material.

## Methods

Briefly, our approach involved combining detailed records of individual food consumption (i.e., dietary surveys) with data on crop pollination requirements and nutrient composition of foods. We evaluated which foods contributed most of the nutrients in the diet and compared scenarios with and without pollinators to estimate the change in nutritional health risks in populations.

### Diet Composition

As a first step in the analysis, we calculated the average proportion of nutrients in the diet that came from pollinator-dependent foods and from different food groups for each country. We obtained dietary recall surveys for children and women in the Central and Eastern Provinces of Zambia, Eastern and Central Uganda, the Zambézia Provence of Mozambique, and the Mymengsingh and Rangpur districts in Bangladesh from collaborators at HarvestPlus (http://www.harvestplus.org). Each survey recorded the age of each individual and the amount of every food consumed in 24 hours by each child or woman. Collaborators at HarvestPlus used a variety of sources to calculate the amount of nutrients in each food item consumed and the data we received already contained this nutrient content information. For all studies, authors accounted for the loss of nutrients due to cooking and collected more than one day of recall for at least a portion of the sampled population. Methods for the collection of dietary recall data and calculations of the nutrient composition of each food can be found in Aresenault et al. [17] for Bangladesh, Hotz et al. [18] for Mozambique, Hotz et al. [19] for Uganda, and Hotz et al. [20] for Zambia.

From these data, we were able to estimate the proportion of daily nutrient intake from each of hundreds of food items. For clarity, we grouped food items into seven categories: dairy; grains; nuts and seeds; fruit; vegetables; meat, poultry, seafood, eggs and insects; and other. The category “other” included oils, flavorings, drinks, candy, honey, and other items that did not fit into other food groups. Within each food category, we further grouped foods according to degree to which pollinators contribute to the yield of each food item (i.e., 0, 5, 25, 65, 95% yield due to pollinators), using estimates of percent yield from published empirical studies (as in, [8], [10]). We then calculated the proportion of total daily nutrient intake that came from each category by individual and day for each nutrient. We averaged these proportions across days for each individual, and then across all individuals in each country. Finally, we re-scaled the proportions so that they added to one for each country for easier comparisons among countries.

### Estimating Risk of Nutrient Deficiency and the Effects of Pollinator Removal

Next, we estimated the potential effects of pollinator declines on risk of nutrient deficiency in the populations surveyed. Specifically, we compared the proportion of the population at risk of developing a nutrient deficiency under the baseline, full pollination scenario to that obtained when assuming complete removal of pollinators. This comparison was made for five age categories (children 1 to less than 4 years, children 4 to less than 9 years, women 19 to less than 51 years who were not pregnant or lactating, lactating women 19 to less than 51 years, and pregnant women 19 to less than 51 years) in each of four countries (Bangladesh, Mozambique, Uganda, and Zambia) across five nutrients (vitamin A, calcium, folate, iron, zinc). There were no individuals aged four to less than nine years old in the Mozambique data and no pregnant women in the Bangladesh data. For all other age categories and countries, sample sizes varied, but were above 150 for all groups except for children four to less than nine years old in Bangladesh (N = 31) and pregnant women in all countries (N<65).

#### Full Pollination Scenario

First, we calculated the total daily intake of vitamin A, calcium, folate, iron, and zinc by individual and day for each of the four countries in the full pollination scenario, by summing the nutrients in the foods consumed by each individual in a day. For this and all subsequent analyses, we used individual food items, not the seven broad food categories used above. The Mozambique and Zambia datasets contained information about whether or not children were currently breastfeeding. For these datasets, only children that were not breastfeeding were included in the analysis.

After calculating total daily intake of nutrients, these data must be adjusted to better estimate the usual nutrient intake distribution of the population. Studies have shown that one or two days of recall do not accurately estimate the usual intake of nutrients. As more days of recall are collected, the mean of the daily intake distribution of a population shifts to slightly higher intakes and the distribution becomes narrower [21], [22]. Several statistical techniques have been proposed to adjust recall data and better estimate the usual intake distribution [21]. We used the Iowa State University method [23], [24] because it is widely accepted and commonly used [21], [25], allows within person variance to vary among individuals [21], uses balanced repeated replication to estimate the standard error for the proportion of the population at risk [21], [26], and is easily implemented in user-friendly, free software (Intake Monitoring, Assessment, and Planning Program (IMAPP) software available at: http://www.side.stat.iastate.edu).

In addition to estimating the usual intake distribution, the IMAPP software estimates the proportion of the population at risk of nutrient deficiencies. It does this in two ways. For vitamin A, zinc, folate, and calcium, IMAPP uses the Estimated Average Requirement (EAR) (i.e., the daily intake of a specific nutrient estimated to meet the needs of 50% of healthy people in a age- and gender-specific groups) cut-point method [27]. Individuals with intakes below the EAR are at risk of nutrient deficiency. For iron, the IMAPP program uses the full probability approach [27] to estimate the proportion of the population at risk because the distribution of iron intakes is often skewed, which violates one of the assumptions of the EAR cut-point method [27]. Because IMAPP uses the EAR cut-point method for most nutrients, estimates of usual intake were performed on age- and gender-specific groups that have identical EAR's. We removed children with missing data for age and removed women with missing information on whether or not they were pregnant or lactating because they could not be placed into age and pregnancy categories for this analysis. In IMAPP, we used harmonized reference intakes, no external variance ratios, and an 18% bioavailability for iron (i.e., the default value in IMAPP). The output of this analysis is the proportion of the population at risk of nutrient deficiency for the five nutrients tested for the full pollination scenario.

#### No Pollinators Scenario

After estimating the proportion of the population at risk of nutrient deficiency under the full pollination scenario, we estimated the effect of complete removal of pollinators on nutrient intake and risk of nutrient deficiency. To do this, we first estimated the percent yield of each food item that was due to pollinators. Most estimates were obtained directly from Klein et al. [8] and Eilers et al. [10]. Klein et al. [8] reviewed the results of empirical studies that quantified the percent yield of crops that was attributed just to animal-pollination for many globally important crops. They assessed the effect of pollination on the production of plant parts, seed production, and breeding and classified crops depending on their need for pollination: essential (production decrease of > = 90% when pollinators were removed), great (40–90% reduction when pollinators were removed), modest (10–40% reduction when pollinators were removed), little (0–10% reduction when pollinators were removed), and no effect of pollinators. Eilers et al. [10] used the categories presented in Klein et al. [8] for their calculations of the percent of global nutrients due to pollinators. For their calculations, Eilers et al. [10] selected the midpoint of the ranges that define the Klein et al. [8] categories. For example, Klein et al. [8] found that the contribution of pollinators to the yield of mangoes was great (40–90%) and Eilers et al. [10] assumed that the percent yield of mangoes due to pollinators was 65 percent.

We used the categories presented in Klein et al. [8] and the percentages assigned by Eilers et al. [10] in our analysis, and included an additional category of 100 percent yield due to pollinators for honey. We also tested the influence of uncertainty in the contribution of pollinators to crop yields on our results for vitamin A intake in children and found that while the specific values for the proportion of the population at risk of nutrient deficiency changed slightly if we used the minimum, midpoint, or maximum percent yield due to pollinators of the categories defined by Klein et al. [8], the significance of the difference between pollination scenarios did not. We used the midpoint of the ranges, as in Eilers et al. [10], for the rest of the analyses and present only those results. Because our analysis focused on consumption, we used only the contribution of pollination to the plant parts that are consumed. In some cases, food items did not fit nicely into one of the Klein et al. [8] or Eilers et al. [10] categories. For these items, we used several rules for assigning percent yield due to pollinators. These rules are described in S1 Table.

After assigning percent yield due to pollinators for each food item, we decreased the amount of each food item consumed per individual per day by the estimated percent yield of each food item due to pollinators. For example, if an individual consumed 100 grams of mango, which included 38 milligrams of vitamin A, we reduced the amount consumed to 35 grams of mango and 13.3 milligrams of vitamin A because the percent yield of mangoes due to pollinators was assumed to be 65 percent. We then calculated the total daily intake of each nutrient for each individual by summing the nutrients in the foods consumed by each individual in a day. We assumed that individuals would not compensate for reduced intake by altering their diet or adding additional foods because it is unclear if and how diets would change and because our objective was to gain a general understanding of the potential contribution of pollinators to nutrient intake and risk of nutrient deficiency.

The usual intake distributions and the proportion of the population below the EAR for each nutrient, country and age category were then estimated with Iowa State University Intake Monitoring, Assessment, and Planning Program (IMAPP) software as described above.

#### Tests for Significance

Finally, we compared the proportion of the population at risk of nutrient deficiency in the full pollination scenario to that of the no pollinators scenario. For vitamin A, calcium, folate, and zinc, IMAPP estimates the proportion of the population below the EAR with standard error. For these nutrients, we tested the significance of the difference between the proportion at risk of nutrient deficiency under the full pollination and no pollinators scenarios with a one-tailed student's t-test in R statistical Software [28]. One-tailed t-tests were used because total nutrient intake for each individual could only stay the same or decrease with pollinator removal. Although one-tailed tests for dependent samples would be most appropriate for this analysis, estimating the usual intake distribution at the population level prevented us from obtaining estimates of usual intake at the level of the individual in both scenarios, which are needed for the t-test for dependent samples. For this reason, we used t-tests for independent samples assuming unequal variances, which require only the mean and standard deviation for each population.

For iron, IMAPP estimates the proportion of the population with inadequate intakes, but does not give a measure of uncertainty around this estimate. However, for all nutrients, IMAPP produces output for the cumulative distribution of nutrient intakes and these estimates are presented with standard error. Therefore, for iron, we graphed the cumulative proportion of the population versus iron intake with +/− two standard errors. If the bands of uncertainty for the full pollination scenario and the no pollinators scenario overlapped at the EAR, we assumed that the proportions of the population at risk in the two scenarios were not significantly different. There were no cases where the bands did not overlap in our results.

Low sample sizes and characteristics of the data (e.g., some individuals with very low intakes on one day of recall and very high intakes on other days) resulted in negative estimates for usual intake variance in some cases (see S2 Table). We were unable to estimate the usual intake distributions with the IMAPP software or compare the proportions of the populations at risk of nutrient deficiency under the two pollination scenarios in these cases.

## Results

**Figure 2:**
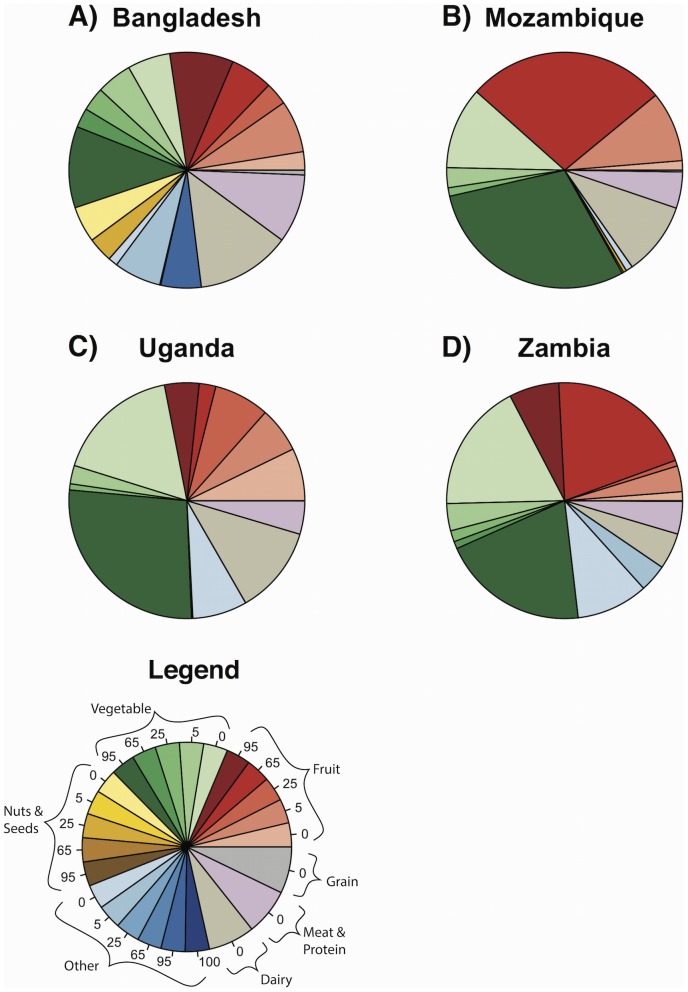
Average proportion of dietary intake of vitamin A from different sources for children 1 to 3 years old. Numbers in the slices of the legend indicate the percent yield due to pollinators. Darker slices are foods that depend heavily on pollinators. “Other” refers to oils, flavorings, drinks, candy, honey, and other items that do not fit into other food groups.

**Figure 3:**
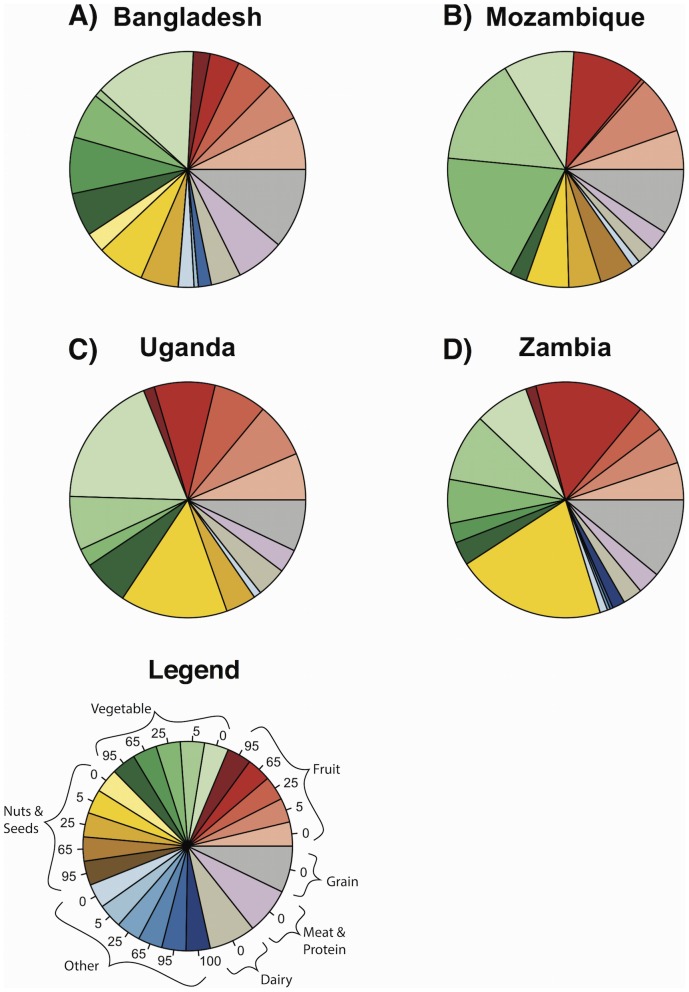
Average proportion of dietary intake of folate from different sources for children 1 to 3 years old. Numbers in the slices of the legend indicate the percent yield due to pollinators. Darker slices are foods that depend heavily on pollinators. “Other” refers to oils, flavorings, drinks, candy, honey, and other items that do not fit into other food groups.

**Figure 4:**
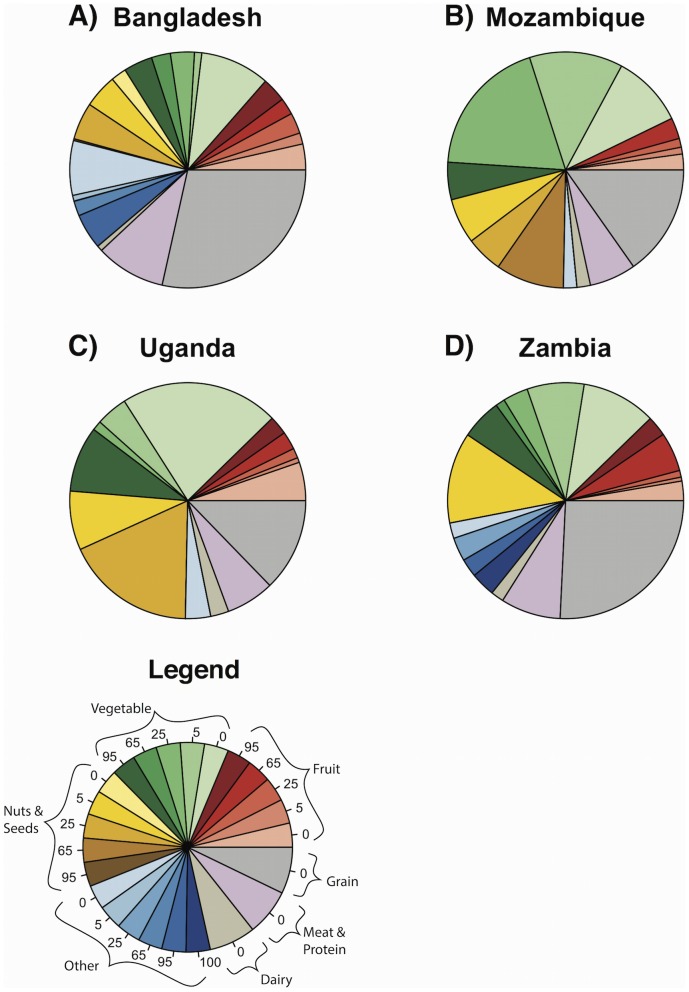
Average proportion of dietary intake of iron from different sources for children 1 to 3 years old. Numbers in the slices of the legend indicate the percent yield due to pollinators. Darker slices are foods that depend heavily on pollinators. “Other” refers to oils, flavorings, drinks, candy, honey, and other items that do not fit into other food groups.

The role of pollinators in determining total nutrient intake varied widely among nutrients and countries (Table 1, Figs. 2, 3, 4). Sixty-nine percent or more of the vitamin A in children's diets came from fruits and vegetables, many of which depend strongly on pollinators (Fig. 2, Table 1, S2 Table). Fruits and vegetables also contributed most of the folate to children's diets, but these plants depended on pollinators to a much lesser degree (Fig. 3, Table 1, S2 Table). The other nutrients appear less dependent on pollinators. Children received a large portion of their calcium from dairy and vegetables, and their iron and zinc from vegetables and grains (Fig. 4, S2 Table); however, dairy and grain products do not depend on animal pollinators [8], and the vegetables providing most of these nutrients did not depend heavily on pollinators (Table 1, S2 Table). For comparison, results for women aged 19 to 50 are presented in S1, S2, S3 Figs., and in S3 Table.

**Table 1:** Average proportion of total nutrient intake by percent yield due to pollinators and country for children 1 to 3 years old.

	% Yield due to Pollinators	Energy	Vitamin A	Calcium	Folate	Iron	Zinc
**Bangladesh**	0	0.74	0.38	0.55	0.48	0.62	0.68
	5	0.05	0.19	0.08	0.14	0.08	0.07
	25	0.08	0.10	0.13	0.17	0.11	0.10
	65	0.04	0.09	0.09	0.12	0.07	0.04
	95	0.08	0.25	0.16	0.10	0.12	0.12
	TOTAL	1.00	1.00	1.00	1.00	1.00	1.00
**Mozambique**	0	0.57	0.29	0.54	0.30	0.37	0.49
	5	0.14	0.13	0.19	0.29	0.20	0.16
	25	0.13	0.02	0.16	0.24	0.25	0.19
	65	0.14	0.27	0.08	0.15	0.12	0.13
	95	0.02	0.30	0.04	0.02	0.05	0.03
	TOTAL	1.00	1.00	1.00	1.00	1.00	1.00
**Uganda**	0	0.68	0.49	0.53	0.40	0.52	0.55
	5	0.14	0.09	0.14	0.30	0.13	0.16
	25	0.10	0.09	0.24	0.14	0.21	0.17
	65	0.04	0.02	0.01	0.08	0.02	0.05
	95	0.04	0.32	0.07	0.08	0.12	0.07
	TOTAL	1.00	1.00	1.00	1.00	1.00	1.00
**Zambia**	0	0.48	0.38	0.41	0.30	0.51	0.51
	5	0.17	0.11	0.17	0.35	0.21	0.24
	25	0.06	0.02	0.14	0.10	0.08	0.10
	65	0.12	0.21	0.13	0.18	0.07	0.03
	95	0.07	0.27	0.11	0.05	0.11	0.10
	100	0.09	0.00	0.04	0.02	0.03	0.02
	TOTAL	1.00	1.00	1.00	1.00	1.00	1.00

**Figure 5:**
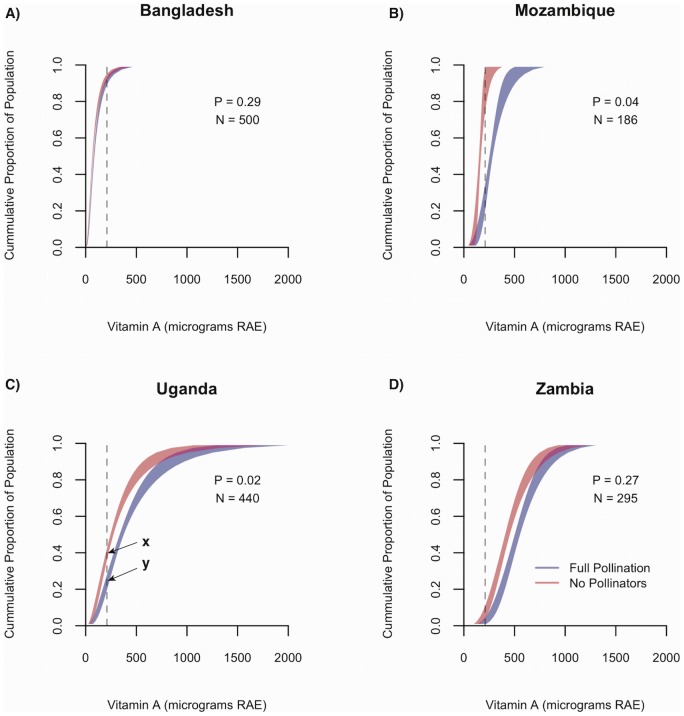
Cumulative distributions of vitamin A intakes for the full pollination scenario (blue) and pollinator removal scenario (pink) for children 1 to 3 years old. Pink and blue bands represent the mean ± one standard error. Vertical dotted lines indicate the estimated average requirement (EAR) for vitamin A intake in this age group (210 µg RAE). The intersection between the pink or blue lines with the vertical dotted line indicates the proportion of the population (y-axis) receiving intakes equal to or less than the EAR (x-axis), and thus, the proportion of the population at risk of nutrient deficiency. For example, for Uganda, 39 percent of the population received equal to or less than the EAR for vitamin A in the no pollinator scenario (x) while 24 percent of the population received equal to or less than the EAR in the full pollinator scenario (y). P is the probability of the null hypothesis that there is no difference between the proportions of the population receiving intakes less than or equal to EAR, N is the sample size, and RAE is retinol activity equivalents.

Based on these results, we hypothesized that pollinators would be most important for nutritional health associated with vitamin A, but less important for nutritional health associated with all other nutrients. To test these hypotheses, we estimated the potential effects of pollinator declines on risk of nutrient deficiency in the populations surveyed. We found that if pollinators were removed, 2 to 56%, 0 to 2%, 0 to 23%, 1 to 5%, and 0.1 to 3% of children in the populations surveyed would become newly at risk of vitamin A, calcium, folate, iron, and zinc deficiencies, respectively (Table 2). For vitamin A in Uganda and Mozambique, this increase was substantial (15% and 56%, respectively) and statistically significant (p<0.05, Fig. 5, Table 2). For folate in Mozambique, the change was also substantial (28%) and marginally significant (p<0.1, Table 2). Folate is a critical nutrient for pre-natal nutrition and is therefore also a concern for pregnant women, but we found only small differences for that group (S4 Table). For all other country-nutrient combinations, increases in risk of nutrient deficiency were minor and not statistically significant (p>0.1, Table 2, S4 Table). These results supported our hypothesis that the risk of vitamin A deficiency is more sensitive to pollinator removal than that of other nutrients.

**Table 2:** Differences in proportion of population at risk of nutrient deficiency between the ‘no pollinators’ and ‘full pollination’ scenarios.

	N	Vitamin A	Calcium	Folate	Iron	Zinc
Bangladesh	500	0.020±0.040	0.001±0.008	0.013±0.028	0.03	0.028±0.038
Mozambique	186	0.560±0.318 *	0±0	0.228±0.164	0.05	0.016±0.039
Uganda	451#	0.150±0.071 *	0.019±0.055	0.041±0.051	0.03	0.009±0.036
Zambia	295	0.050±0.079	0.008±0.043	−0.002±0.068	0.01	0.001±0.010

Data are differences in means ± one standard error of that difference, for children 1 to 3 years old. N is the sample size. See Methods section for explanation of missing standard errors for iron and Table S4 for the proportion of populations at risk in each scenario.

*P<0.05 for one-tailed t-test of difference between means.

# N = 451 for all nutrients except vitamin A where N = 440.

## Discussion

Our results suggest a highly variable but important role for pollinators in human nutrition in the developing world. In four countries and across five nutrients, we found that 0 to 56% of populations would become newly at risk of nutritional deficiency if pollinators were removed. The importance of pollinators to human nutrition therefore depends critically on the composition of local diets, and cannot be reliably predicted from global commodity analyses.

While the risk of nutrient deficiency did not change substantially for most nutrients when pollinators were removed, the increased risk we estimated for vitamin A carries concerning public health implications. Each year, vitamin A deficiency causes an estimated 800,000 deaths in women and children, including 20–24% of child mortality from measles, diarrhea and malaria and 20% of all-cause maternal mortality. It is estimated to roughly double the risk of mortality from common conditions like measles, diarrhea, and malaria while increasing the risk of maternal mortality 4.5 times [29].

The pattern of results for vitamin A also indicates that nutritional vulnerability to pollinator decline depends not only on the specific sources of nutrients in the diet and their pollinator dependence, but also on the total amount of nutrients being consumed. In Bangladesh, for example, removal of pollinators had little effect on the risk of vitamin A deficiency because most individuals were already malnourished (Fig. 5A). In Zambia, on the other hand, children received much of their vitamin A from pollinator-dependent fruits and vegetables, but had daily vitamin A intakes well above the EAR. The decrease in intake caused by pollinator removal was therefore not enough to significantly increase risk of nutrient deficiency (Fig. 5D).

More generally, our findings make clear that estimating the health effects of pollinator decline requires modeling the full causal chain linking pollinators to nutrient deficiency (Fig. 1). The wide differences we find among populations for vitamin A (Table 2) suggest that global data on crop and nutrient production (e.g., [10]) are insufficient for predicting the effects of pollinator declines on human health. Diets vary substantially among populations, and some may not depend on pollinator-dependent crops for nutrients. Furthermore, individuals in our study consumed many locally harvested foods (e.g., leaves, flowers, shoots, insects, and mammals from natural areas) that are not included in global commodity analyses. Individual consumption choices and resulting diets appear to be as important to the role of pollinators in human nutrition as the production and pollination ecology of the crops themselves.

Although this study provides an important first step in understanding the importance of pollinators for nutritional health, we made several simplifying assumptions that must be considered when interpreting our results. First, we assumed a complete removal of pollinators, a possible but unlikely scenario given current trends in pollinator abundance [6]. Second, we assumed that individuals would not compensate for reduced intake by altering their diets or substituting other foods. This is also unlikely, but substitutes would probably be less pollinator-dependent than the foods they replace. Finally, we assumed no interactions among nutrients. The absorption of some nutrients may depend on the presence or absence of others [30], [31], but most nutrient interactions are not yet well understood. Relaxing the first two assumptions would tend to lower our estimates of the role of pollinators in determining health risks, while the effects of the third assumption are difficult to predict and would differ among nutrients.

Three sources of uncertainty are also important to consider. First, there is uncertainty in the contribution of pollinators to crop yields (as presented in, [8]). We tested the effects of this uncertainty [8] on a subset of our results for vitamin A and found no qualitative change in our results. Second, the datasets we analyzed included only two days of recall. While having multiple days of recall allowed us to estimate within and between person variation and use those sources of variation to estimate usual nutrient intake distributions [23], [24], two days of recall are unlikely to capture the true nature of variation in nutrient intake for individuals, especially in areas where diets are highly seasonal [22]. Third, uncertainty in the estimate of usual intake distributions is determined by sample sizes, which could limit our power to detect change in risk. In a few cases, we saw a relatively large effect size that was not significant (e.g., folate in Mozambique (Table 2)), which could indicate a lack of power.

Our results from four countries and five nutrients indicate that the loss of pollinators is most likely to affect nutritional health when: (1) individuals receive the majority of their nutrients from fruits and vegetables that depend heavily on pollinators, (2) individuals are neither severely deficient nor receiving nutrient intakes well above the EAR, (3) individuals are unable to substitute other foods to fully replace nutrients lost by the removal of pollinator-dependent foods from the diet, and (4) individuals do not have access to nutrient supplements, fortified foods, or targeted nutrition programs. The links between pollinators and human nutrition are clearly complex [11], and the global health importance of pollinator decline depends on where and how often these four conditions co-occur. Expanding dietary intake surveys to be nationally representative, address additional vulnerable countries, sample more people within each country, and capture seasonality would help most to identify populations most vulnerable to pollinator declines.

Of course, the potential health effects of ecosystem change extend far beyond pollination [3]. Forest clearing and other development activities, for example, have caused water pollution, increased exposure to vector-borne disease, and reduced access to nutritious foods found in forested areas [4]. Understanding these effects can inform and connect conservation and public health policies, but will require novel synthesis among ecological, behavioral, and epidemiological science (Fig. 1).

## Supporting Information

S1 Fig **Average proportion of dietary intake of vitamin A from different sources for women 19 to 50 years old (including pregnant and lactating women).** Numbers in the slices of the legend indicate the percent yield due to pollinators. Darker slices are foods that depend heavily on pollinators. “Other” refers to oils, flavorings, drinks, candy, honey, and other items that do not fit into other food groups.
(TIF)

S2 Fig **Average proportion of dietary intake of folate from different sources for women 19 to 50 years old (including pregnant and lactating women).** Numbers in the slices of the legend indicate the percent yield due to pollinators. Darker slices are foods that depend heavily on pollinators. “Other” refers to oils, flavorings, drinks, candy, honey, and other items that do not fit into other food groups.
(TIF)

S3 Fig **Average proportion of dietary intake of iron from different sources for women 19 to 50 years old (including pregnant and lactating women).** Numbers in the slices of the legend indicate the percent yield due to pollinators. Darker slices are foods that depend heavily on pollinators. “Other” refers to oils, flavorings, drinks, candy, honey, and other items that do not fit into other food groups.
(TIF)

S1 Table **Rules used to assign percent yield due to pollinators for all food items in the diet.**
(DOCX)

S2 Table **Average proportion of total daily energy and nutrient intake coming from major food groups by percent yield due to pollinators for children 1 to 3 years old.** N is sample size. See ‘Diet Composition’ section in the ‘Methods’ of the main text for an explanation of the calculation of these data.
(XLSX)

S3 Table **Average proportion of total daily energy and nutrient intake coming from major food groups by percent yield due to pollinators for women 19 to 50 years old (including pregnant and lactating women).** N is sample size. See ‘Diet Composition’ section in the ‘Methods’ of main text for an explanation of the calculation of these data.
(XLSX)

S4 Table **Risk of nutrient deficiency estimates across nutrients, age groups, and countries.** See the main text and ‘Methods’ in the main text for explanation of the absence of standard error for the proportion of the population below the EAR for iron. N is the sample size and does not include individuals with missing data for age categories. See ‘Methods’ for handling of missing data.
(XLSX)
