# Supplementary figures and images for: Do Pollinators Contribute to Nutritional Health?
Source: PLoS One. 2015 Jan 9;10(1):e114805. doi: 10.1371/journal.pone.0114805 (PMC4289064; doi:10.1371/journal.pone.0114805)

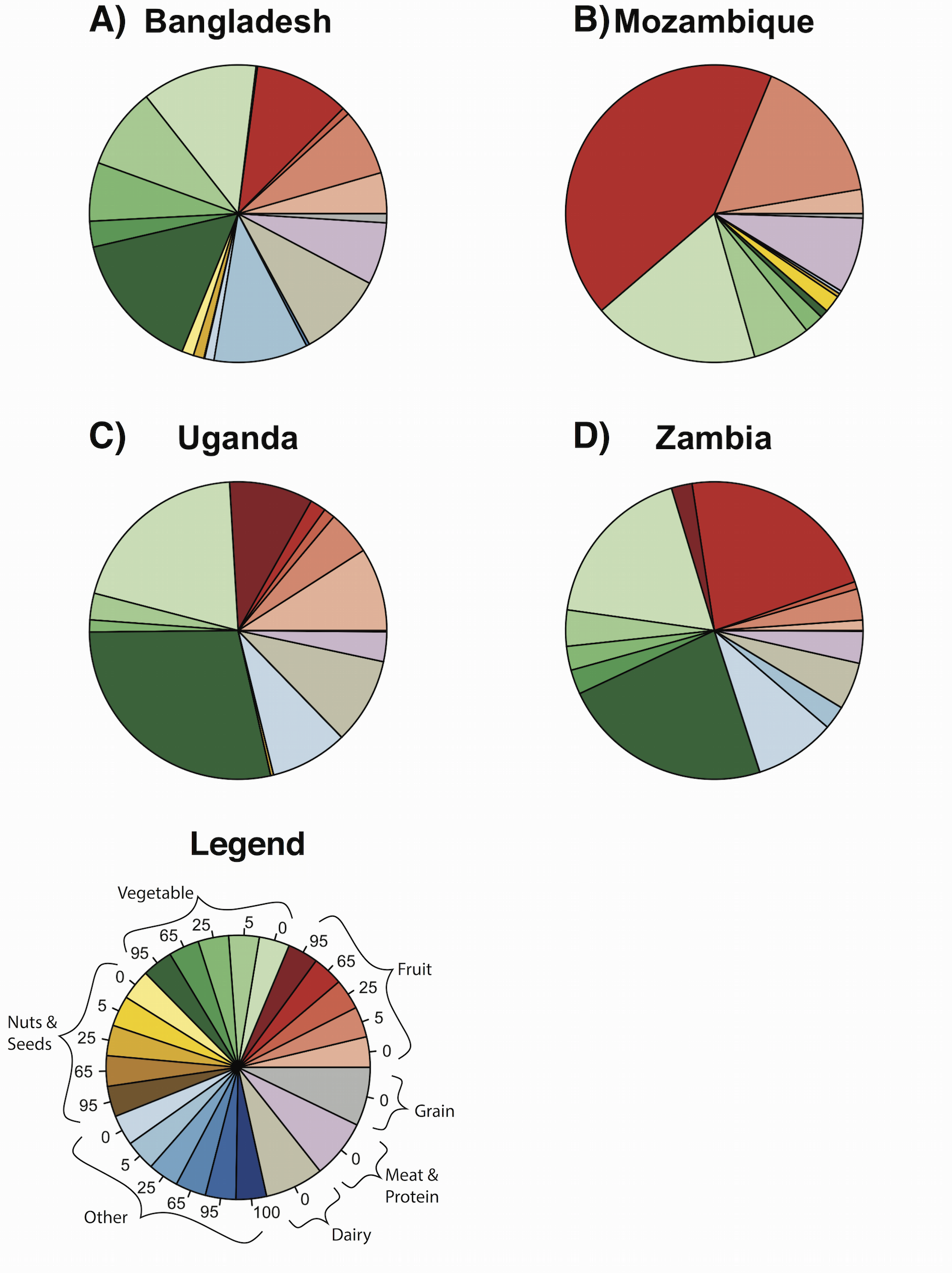

Supplement: S1 Fig — Average proportion of dietary intake of vitamin A from different sources for women 19 to 50 years old (including pregnant and lactating women). Numbers in the slices of the legend indicate the percent yield due to pollinators. Darker slices are foods that depend heavily on pollinators. “Other” refers to oils, flavorings, drinks, candy, honey, and other items that do not fit into other food groups. (TIF) [file pone.0114805.s001.tif]

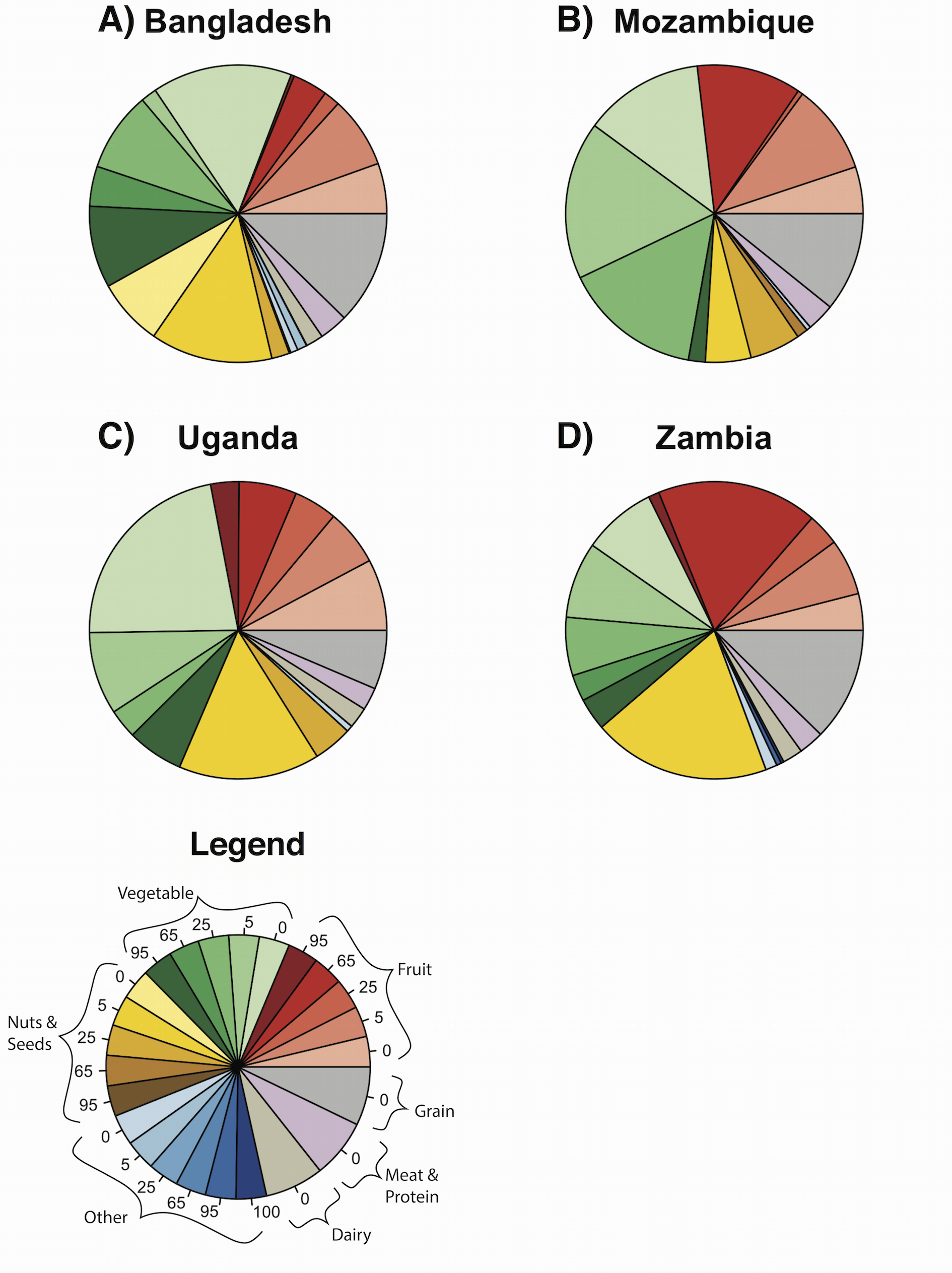

Supplement: S2 Fig — Average proportion of dietary intake of folate from different sources for women 19 to 50 years old (including pregnant and lactating women). Numbers in the slices of the legend indicate the percent yield due to pollinators. Darker slices are foods that depend heavily on pollinators. “Other” refers to oils, flavorings, drinks, candy, honey, and other items that do not fit into other food groups. (TIF) [file pone.0114805.s002.tif]

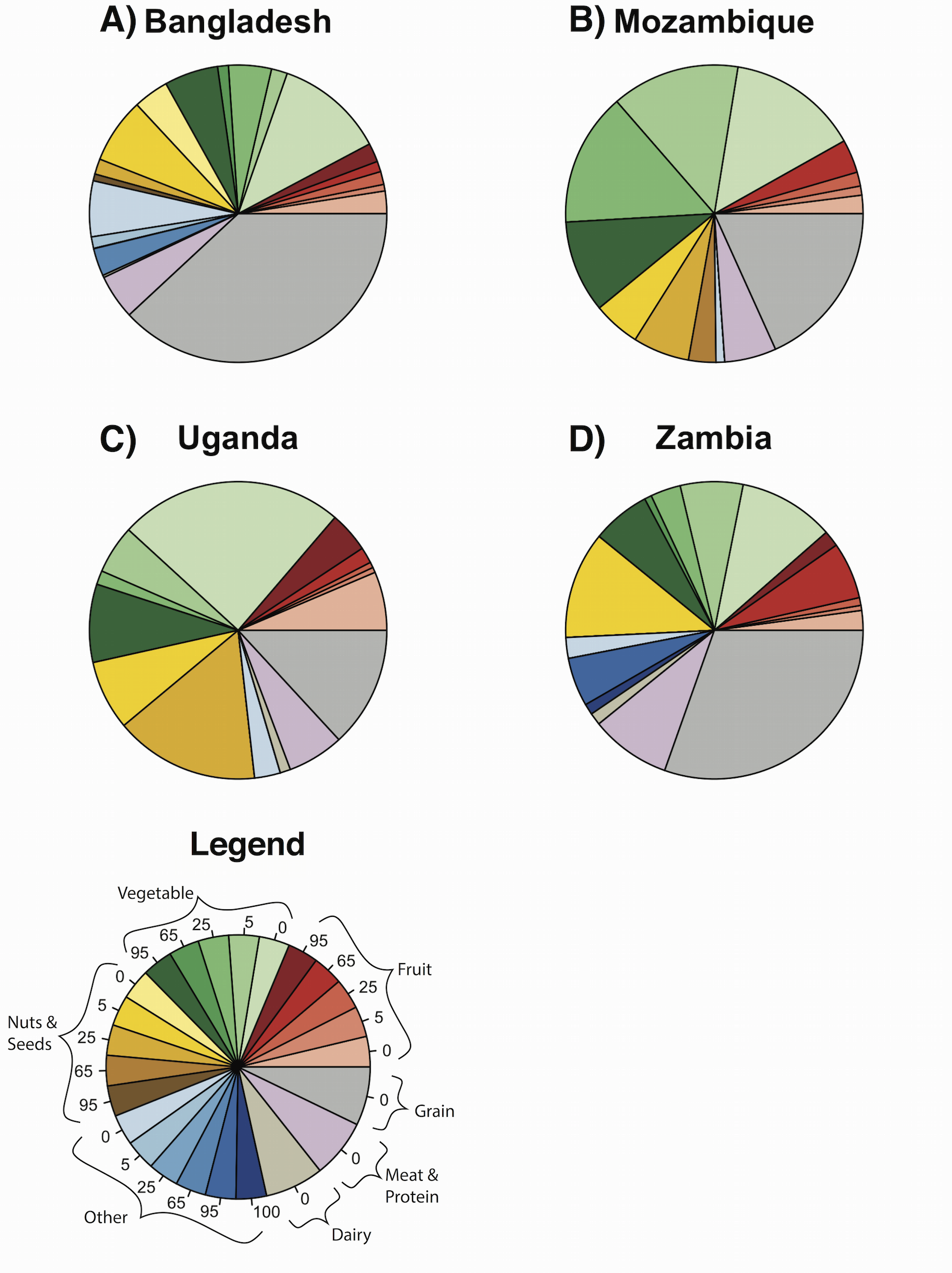

Supplement: S3 Fig — Average proportion of dietary intake of iron from different sources for women 19 to 50 years old (including pregnant and lactating women). Numbers in the slices of the legend indicate the percent yield due to pollinators. Darker slices are foods that depend heavily on pollinators. “Other” refers to oils, flavorings, drinks, candy, honey, and other items that do not fit into other food groups. (TIF) [file pone.0114805.s003.tif]
