# Supplementary material for: Do Pollinators Contribute to Nutritional Health?
Source: PLoS One. 2015 Jan 9;10(1):e114805. doi: 10.1371/journal.pone.0114805 (PMC4289064; doi:10.1371/journal.pone.0114805)
Supplement: S1 Table — Rules used to assign percent yield due to pollinators for all food items in the diet. (DOCX) [file pone.0114805.s004.docx]

1Table S1.

| **Description** |  | **Rule** |
| --- | --- | --- |
| **A. Single Food Item** |  |  |
|  |  |  |
| 1. Item listed in Klein et al. ([8](#_ENREF_9)) |  |  |
|  |  |  |
| a. Contribution to pollination known |  |  |
|  |  |  |
| i. Pollination contributes to edible portion of plant |  | Use category assigned to item in Klein et al. ([8](#_ENREF_9)) and select the midpoint of the percent yield increase range for that category (as in Eilers et al. ([10](#_ENREF_11))) |
|  |  |  |
| ii. Pollination contributes to breeding or non-edible seeds |  | Assume no contribution of pollination to yield (percent yield due to pollination = 0) |
|  |  |  |
| b. Contribution to pollination unknown |  | Assume no contribution of pollination to yield (percent yield due to pollination = 0) |
|  |  |  |
| 2. Item not listed in Klein et al. ([8](#_ENREF_9)) |  |  |
|  |  |  |
| a. Item is leaf, flower, tuber, mushroom, branch, or shoot |  | Assume no contribution of pollination to yield (percent yield due to pollination = 0) |
|  |  |  |
| b. Item is not leaf, flower, tuber, mushroom, branch, or shoot |  |  |
|  |  |  |
| i. Contribution to pollination found in literature |  | Use percent yield due to pollination from the literature to place the item into one of the categories described by Klein et al. ([8](#_ENREF_9)). Then select the midpoint of the percent yield increase range for that category (as in Eilers et al. ([10](#_ENREF_11))) |
|  |  |  |
| ii. Contribution to pollination not found in literature |  | Assume no contribution of pollination to yield (percent yield due to pollination = 0) |
|  |  |  |
| **B. Broad Food Category with Multiple Varieties** |  |  |
|  |  |  |
| 1. Category comprised of items with same percent yield due to pollination as listed in Klein et al. ([8](#_ENREF_9)). |  | Use category assigned to the items in Klein et al. ([8](#_ENREF_9)) and select the midpoint of the percent yield increase range for that category (as in Eilers et al. ([10](#_ENREF_11))) |
|  |  |  |
| 2. Components of the category do not have the same percent yield due to pollination |  | Use the category for the item with the lowest percent contribution of pollination and select the midpoint of the percent yield increase range for that category (as in Eilers et al. ([10](#_ENREF_11))) |
|  |  |  |
| 3. Components of the category are unclear |  | Assume no contribution of pollination to yield (percent yield due to pollination = 0) |
|  |  |  |
| **C. Food Comprised of Multiple Ingredients** |  |  |
|  |  |  |
| 1. Ingredients listed in dataset |  |  |
|  |  |  |
| a. There is one major ingredient and the yield of that ingredient is sensitive to pollination |  | Find category assigned to major ingredient in Klein et al. ([8](#_ENREF_9)). Select category below this category (lower contribution of pollination) & use midpoint of the percent yield increase range for the lower category |
|  |  |  |
| b. There are 2 or 3 major ingredients and the yields of those ingredients are sensitive to pollination |  | Find categories assigned to major ingredients in Klein et al. ([8](#_ENREF_9)). Select one category of these with the lowest contribution of pollination to yield. Select category below this category (lower contribution of pollination) & use midpoint of the percent yield increase range for the lower category |
|  |  |  |
| c. None of the major ingredients are sensitive to pollination |  | Assume no contribution of pollination to yield (percent yield due to pollination = 0) |
|  |  |  |
| 2. Ingredients not listed in dataset |  |  |
|  |  |  |
| a. Typical ingredients found in the literature |  | Follow rules for C.1 above |
|  |  |  |
| b. Typical ingredients not found in the literature |  | Assume no contribution of pollination to yield (percent yield due to pollination = 0) |
|  |  |  |
| **D. Other** |  |  |
|  |  |  |
| 1. Fruit Juice |  |  |
|  |  |  |
| a. Juice is 100% fruit |  | Use category assigned to fruit in Klein et al. ([8](#_ENREF_9)) and select the midpoint of the percent yield increase range for that category (as in Eilers et al. ([10](#_ENREF_11))) |
|  |  |  |
| b. Percent real juice not listed or unclear |  | Assume no contribution of pollination to yield (percent yield due to pollination = 0) |
|  |  |  |
